# Renal Autologous Cell Therapy to Stabilize Function in Diabetes-Related Chronic Kidney Disease: Corroboration of Mechanistic Action With Cell Marker Analysis

**DOI:** 10.1016/j.ekir.2022.04.014

**Published:** 2022-04-21

**Authors:** Joseph Stavas, Guido Filler, Deepak Jain, John Ludlow, Joydeep Basu, Richard Payne, Emily Butler, Maria Díaz-González de Ferris, Tim Bertram

**Affiliations:** 1 ProKidney, LLC, Raleigh, North Carolina, USA; 2 Departments of Paediatrics, Medicine, Pathology and Laboratory Medicine, University of Western Ontario, London, Canada; 3 Schulich School of Medicine and Dentistry, University of Western Ontario, London, Canada; 4 Lilibeth Caberto Kidney Clinical Research Unit, London, Ontario, Canada; 5 Department of Pediatrics, University of North Carolina at Chapel Hill, Chapel Hill, North Carolina, USA

**Keywords:** cell-based therapy, chronic kidney disease, estimated glomerular filtration rate, selected renal cells, type 2 diabetes mellitus

## Abstract

**Introduction:**

Chronic kidney disease (CKD) is a worldwide disease without cure. Selected renal cells (SRCs) can augment kidney function in animal models. This study correlates the phenotypical characteristics of autologous homologous SRCs (formulated product called Renal Autologous Cell Therapy [REACT]) injected into patients’ kidneys with advanced type 2 diabetes-related CKD (D-CKD) to clinical and laboratory findings.

**Methods:**

A total of 22 adults with type 2 D-CKD underwent a kidney biopsy followed by 2 subcortical injections of SRCs, 7 ± 3 months apart. There were 2 patients who had only 1 injection. We compared annualized estimated glomerular filtration rate (eGFR) slopes pre- and post-REACT injection using the 2009 CKD-EPI formula for serum creatinine (sCr) and the 2012 CKD-EPI Creatinine-Cystatin C equation and report clinical/laboratory changes. Fluorescent Activated Cell Sorting (FACS) Analysis for renal progenitor lineages in REACT and donor vascular endothelial growth factor A (VEGF-A) analysis were performed. Longitudinal parameter changes were analyzed with longitudinal linear mixed effects model.

**Results:**

At baseline, the mean diabetes duration was 18.4 ± 8.80 years, glycated hemoglobin (Hgb) was 7.0 ± 1.05, and eGFR was 40.3 ± 9.35 ml/min per 1.73 m^2^ using the 2012 CKD-EPI cystatin C and sCr formulas. The annualized eGFR slope (2012 CKD-EPI) was −4.63 ml/min per 1.73 m^2^ per year pre-injection and improved to −1.69 ml/min per 1.73 m^2^ per year post-injection (*P* = 0.015). There were 7 patients who had an eGFR slope of >0 ml/min per 1.73 m^2^ postinjection. SRCs were found to have cell markers of ureteric bud, mesenchyme cap, and podocyte sources and positive VEGF. There were 2 patients who had remote fatal adverse events determined as unrelated with the biopsies/injections or the REACT product.

**Conclusion:**

Our cell marker analysis suggests that SRCs may enable REACT to stabilize and improve kidney function, possibly halting type 2 D-CKD progression.

## Graphical abstract

**Figure:**
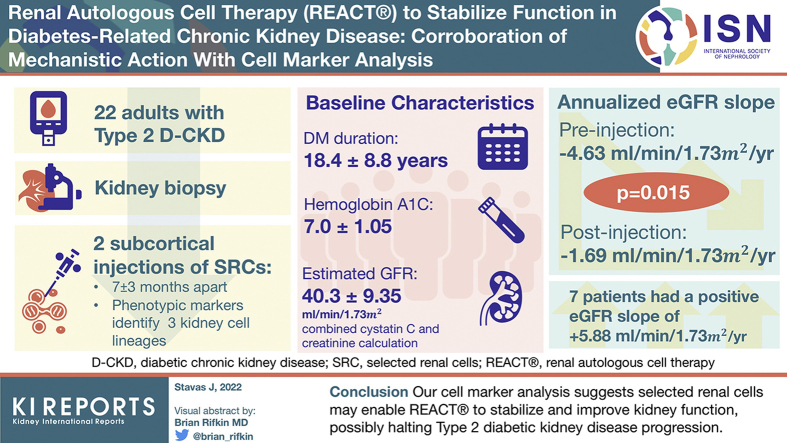

Approximately 37 million US adults have CKD, a major public health concern, as it is a progressive condition that culminates in end-stage kidney disease (ESKD).^1^^,^^2^ The economic/societal costs of CKD are substantial.^2^^,^^3^ In 2018, ESKD represented 7.2% US Medicare spending.^4^ CKD is underreported with inconsistency in kidney disease testing.^5^ Large registry-based studies indicate that D-CKD is the most common cause of ESKD.^6^

The pathomorphologic sequence of nephron loss with glomerular decapitation and progressive tubular fibrosis has been described in diabetic nephropathy.^7^ Once nephron loss begins, no new organoids can be formed.^7^ There is no cure for CKD and treatments are small molecules targeting biochemical pathways in the kidney to affect related comorbidities; however, the underlying glomerular and tubulointerstitial dysfunction remains unaltered. Cell-based therapies are a promising treatment, and in preclinical CKD models, they reduce inflammation and fibrosis, resulting in kidney function stabilization.^8^^,^^9^

In animal models, we revealed that isolated and expanded SRCs injected into diseased kidneys augment kidney function and improve survival.^10^^,^^11^ On the basis of this evidence, we are conducting Federal Drug Administration-approved human clinical trials.^12^, ^13^, ^14^ Elucidating the mechanism of how SRC injections restore kidney function in humans is key to understanding the potential of this therapy for stabilizing CKD.

SRCs are the bioactive product in REACT derived from the patients’ own kidney cells. The REACT product is an admixture principally of epithelial cells from the proximal tubules and glomeruli and smaller numbers of other cell subpopulations, such as interstitial cells, collectively called SRCs.^15^ Animal models suggest that kidney restoration with SRC-based therapies may mirror fetal kidney development.^11^ REACT may afford the diseased kidney components of the developmental pathway to initiate a cascade of events that halt disease progression and/or restore kidney function.^16^^,^^17^ We present early findings in a subpopulation of 22 patients with moderate to advanced type 2 D-CKD who received REACT as a part of our larger ongoing clinical trial (NCT02836574). We describe change in kidney function and characterize the progenitor cell lines of the REACT product with FACS analysis of membrane-bound nuclear transcription factors, representing cap mesenchyme, ureteric bud, and glomerular lineages (Supplementary Appendix 1), correlating levels of these factors with observed kidney outcomes. We also evaluated secretion of VEGF-A in conditioned media sourced from human SRCs. This is a proof-of-concept clinical trial suggesting that SRC therapy may enable kidney function stabilization through neo kidney-like tissue.^16^

## Methods

### Study Population

We enrolled 30- to 80-year-old patients with type 2 D-CKD from multiple institutions across the USA, who had an eGFR of 20 to 50 ml/min per 1.73 m^2^ and had consented to the parent study.^18^ The diagnosis of type 2 D-CKD was made by clinical diagnosis and did not require histopathologic evidence. Their comorbidities were managed with standard of care, and sodium-glucose transport protein 2 receptor blockade treatment was not a contraindication. Except for diabetes-related conditions, patients with incapacitating comorbidities were excluded. Our cohort only included selected REACT-treated patients from the parent study who consented to this publication and whose SRCs were characterized by FACS.

### Biopsy and REACT Injection Procedures

All patients underwent an image-guided standard percutaneous kidney biopsy to isolate and expand kidney cells, creating autologous homologous SRCs. The SRCs were formulated in thermolabile hydrogel to manufacture a fresh REACT product. The first computed tomography (CT)-guided percutaneous injection of patient’s autologous SRCs occurred approximately 3 months after the biopsy, and the second percutaneous injection occurred 7 ± 3 months after the first injection (based on clinical observations and trial protocols).^18^^,^^19^ There were 2 patients who received only 1 injection. The REACT dosing was patient specific, based on kidney volume calculated by magnetic resonance imaging. The cumulative REACT dose ranged from 8 to 16 ml (8.0–16 × 10^8^ SRCs) for 20 patients receiving 2 injections and 4.5 to 5.5 ml (4.5–5.5 × 10^8^ SRCs) for 2 patients who received only 1 injection. All procedures were under conscious sedation protocols using i.v. midazolam and fentanyl and same-day-admission outpatient visits.

### Clinical and Laboratory Data

Clinical and laboratory data were collected at the time of the first REACT injection and every 3 months for 1 year after the first injection. Biochemical measures included electrolytes Hgb, sCr, urea nitrogen, phosphorus, calcium, potassium, bicarbonate, glycated Hgb, log-transformed intact parathyroid hormone, and log-transformed urinary albumin/sCr ratio (UACR). eGFR was estimated using the 2009 CKD-EPI formula for IDMS traceable serum sCr alone and the 2012 CKD-EPI formula for sCr and cystatin C (nephelometry with certified reference materials).^20^^,^^21^ We analyzed the cohort before and after they received the first REACT injection, establishing their eGFR slope pre- and post-intervention. Patients were classified based on their annualized eGFR slopes (in ml/min per 1.73 m^2^ per year) as low responders (<0), moderate responders (≥0 and ≤2), and high responders (>2), but for analysis purposes, we combined the moderate and high responder groups and compared them with low responders.

### Serious Adverse Events

Serious adverse events (SAEs) were determined by event seriousness and intensity per reporting from the Medical Dictionary for Regulatory Activities version 23.0 into Preferred Terms and System Organ Classes.

### Ethics and Trial Registration

The trial was approved by the research ethics board of the participating centers and registered at http://clinicaltrials.gov/ct2/show/NCT02836574. The first patient consented to the study on March 9, 2017. The trial protocol was approved by each site’s Institutional Review Board or Ethics Committee on human research and the patients provided written informed consent to perform cellular analyses of their SRCs.

### Phenotypic Marker Analysis of SRCs

FACS analysis determined whether the SRCs contained markers associated with cap mesenchyme, ureteric bud, and glomeruli. The analysis of these proteins, which are mostly transcription factors to describe SRC phenotype (Supplementary Appendix 1), was performed as described by Burnette and Bruce in 2013.^22^

### VEGF-A Enzyme-Linked Immunosorbent Assay

Three different human SRC cultures (TCHK-030, 031, 032) produced identically to the REACT product from cadaveric donor tissue with conditioned media, and negative control media were analyzed. Individual and average VEGF concentrations were measured at 1-month intervals in triplicate using the Human VEGF Quantikine enzyme-linked immunosorbent assay (R&D Systems), according to the manufacturer’s protocol.

### Statistics

We used simple descriptive statistics for screening demographic and laboratory measures. Normally distributed parameters (determined by Shapiro-Wilk normality test) were expressed as mean ± SD. Not normally distributed parameters were log-transformed. Longitudinal data were compared using a longitudinal linear mixed effect models with a correlated random intercept and slope (for time).^23^ Patient identifier was a random effect, time was a fixed effect, and eGFR was the dependent variable. This analysis was summarized as the annualized slope: the average change in eGFR over 1 year time. To perform this analysis, the preinjection annualized slope used all measurements from screening until and including day of injection, as this measurement was taken before the injection. The postinjection annualized slope used all measurements from the day after injection to the end of each patient’s follow-up. All retest measurements and unscheduled visits were included in the analysis for both the pre- and post-injection analyses. We did not perform a power calculation, as the sample size was small. Some patients had a 24-hour UACR rather than a random sample, and here the random UACR was imputed.

## Results

### Patients

A total of 28 patients were eligible for this interim analysis. Six did not consent to have their data published. In the final sample of 22 patients, 2 received only 1 injection of REACT; after their first injection, 1 patient commenced on clopidogrel and the second one had uncontrolled hypertension. In the remaining 20 patients with 2 REACT injections, blood pressure did not change (data not shown). Because only 3 patients were on phosphate binders, 2 on erythropoietin supplementation, and 4 on potassium binders, we did not analyze the impact of REACT on phosphorus, Hgb, or potassium levels. Of the 22 patients, 10 were treated with an angiotensin-converting enzyme inhibitor, 13 patients received an angiotensin II receptor blocker, and 3 patients were treated with sodium-glucose transport protein 2 inhibitors.

Laboratory values at screening are depicted in Table 1, with a mean CKD-EPI 2009 eGFR of 37.3 ± 8.91 ml/min per 1.73 m^2^ and a mean glycated Hgb of 7.0 ± 1.05%. Consistent with the expected decline, the eGFR dropped to 33.0 ± 8.91 ml/min per 1.73 m^2^ at the time of the first REACT injection. Using linear mixed effect model, the annualized eGFR slope improved significantly between pre-injection (-3.98 ml/min per 1.73 m^2^ per year) and post-injection of REACT (-1.27 ml/min per 1.73 m^2^ per year) in the full cohort (*P* = 0.032).

**Table 1:** Patient characteristics at the time of screening

Characteristic at the time of screening *n =* 22, 17 males	Value [mean ± (SD)]
Age	66.0 (9.04) yr
BMI	33.9 (5.87) kg/m^2^
Duration of diabetes	18.4 (8.80) yr
Race	82% White, 4.5% African American, 4.5% Native American, 9% others
eGFR based on 2009 CKD-EPI using sCr only	37.3 (8.91) ml/min per 1.73 m^2^
eGFR based on 2012 CKD-EPI (combined sCr and cystatin C)	40.3 (9.35) ml/min per 1.73 m^2^
Log(urinary albumin/creatinine ratio, mg/g)	5.7 (2.00)
Hemoglobin	12.4 (1.57) g/dl
Serum calcium	9.3 (0.55) mg/dl
Serum phosphate	3.7 (0.66) mg/dl
Log(intact parathyroid hormone, pg/ml)	4.1 (0.58)
Potassium	4.7 (0.51) mmol/l
Bicarbonate	20.4 (2.70) mmol/l
Hemoglobin A1c	7.0 (1.05)%

BMI, body mass index; CKD-EPI, Chronic Kidney Disease Epidemiology Collaboration; eGFR, estimated glomerular filtration rate; sCR, serum creatinine.

**Figure 1:**
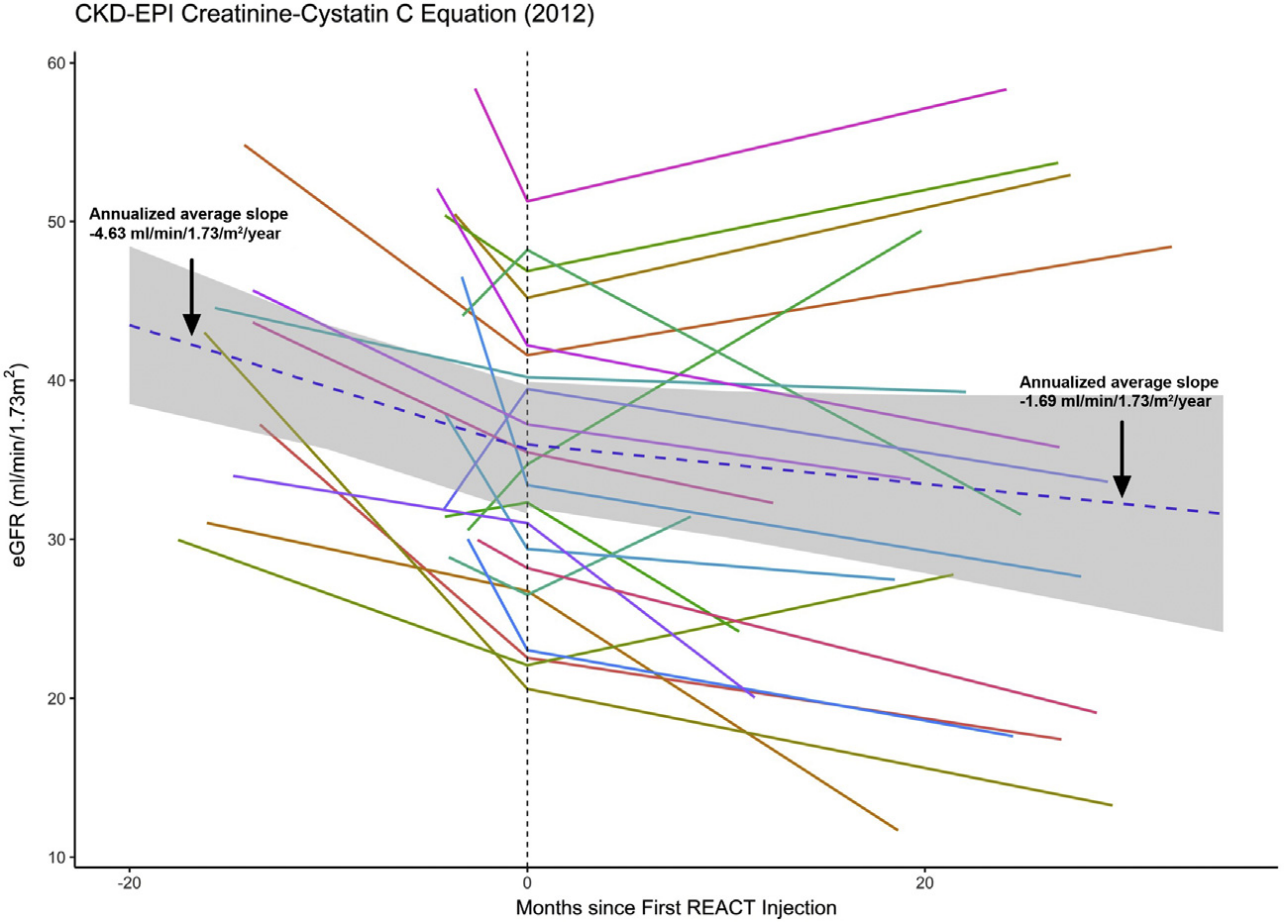
Response of eGFR (creatinine and cystatin C, 2012 CKD-EPI) to SRC injection. Although the overall group had a stabilization of the eGFR decline, 7 patients had a sustained increase of their eGFR. Day 0 represents the date of the first injection. Using linear mixed effects model, the annualized eGFR slope significantly improved from −4.63 ml/min per 1.73 m^2^ per year to −1.69 ml/min per 1.73 m^2^ per year (*P* = 0.015). CKD-EPI, Chronic Kidney Disease Epidemiology Collaboration; eGFR, estimated glomerular filtration rate; REACT, Renal Autologous Cell Therapy; SRC, selected renal cell.

Using the 2012 CKD-EPI equation based on both sCR and cystatin C, the annualized eGFR slope improved from -4.63 ml/min per 1.73 m^2^ per year preinjection and improved to -1.69 ml/min per 1.73 m^2^ per year (*P* = 0.015) postinjection (see Figure 1). Median follow-up was 24.3 (interquartile range 18.8, 27.7) months. Linear mixed effect model analyses (see Table 2) revealed that REACT slowed the increase of log parathyroid hormone (*P* = 0.04) and UACR (*P* = 0.001).

**Table 2:** Linear mixed effects model analysis of various clinical parameters comparing preinjection and postinjection annualized slope

Study parameter	Preinjection annualized slope	Postinjection annualized slope	*P* value
eGFR (CKD-EPI creatinine 2009 equation)	-3.98 ml/min per 1.73 per m^2^ per yr	-1.27 ml/min per 1.73 per m^2^ per yr	0.032
eGFR (CKD-EPI creatinine-cystatin C 2012 equation)	-4.63 ml/min per 1.73 per m^2^ per yr	-1.69 ml/min per 1.73 per m^2^ per yr	0.015
Serum creatinine	0.14 mg/dl per yr	0.18 mg/dl per yr	0.54
Cystatin C	0.1 mg/l per yr	0.13 mg/l per yr	0.59
BUN	4.74 mg/dl per yr	0.72 mg/dl per yr	0.07
Phosphorus	0.12 mg/dl per yr	0.15 mg/dl per yr	0.82
Calcium	0.02 mg/dl per yr	-0.12 mg/dl per yr	0.10
Serum potassium	0.02 mEq/l per yr	0.00 mEq/l per yr	0.83
Serum Bicarbonate	-0.47 mEq/l per yr	0.33 mEq/l per yr	0.13
Log (PTH)	0.8	0.19	0.04
Hemoglobin	-0.09 g/dl per yr	-0.07 g/dl per yr	0.93
Log (UACR)	-0.42	0.23	0.001

BUN, bound urea nitrogen; CKD-EPI, Chronic Kidney Disease Epidemiology Collaboration; eGFR, estimated glomerular filtration rate; PTH, parathyroid hormone; sCR, serum creatinine; UACR, urinary albumin/sCr ratio.

There were 7 patients (32%) who had a positive postinjection eGFR slope (3 were “moderate responders” and 4 were “high responders”). Annualized slope of the moderate/high responders was 5.88 ml/min per 1.73 m^2^ per year, compared with the low responders which was -3.96 ml/min per 1.73 m^2^/yr. On average, the moderate/high responders received their second injection 7.8 months after their first (compared with 6.2 months in the low responders’ group). Their baseline (last pre-injection measurement) average eGFR was 38.9 ± 6.69 ml/min per 1.73 m^2^, compared with the remaining “low responders”: 30.2 ± 7.80 ml/min per 1.73 m^2^. These 7 patients with a positive eGFR slope had an average log UACR of 4.6 ± 2.005 mg/mg, compared with other 15 patients with 6.4 ± 1.56 mg/mg at baseline. High/moderate responders received a mean REACT cumulative dose of 10.4 ± 2.84 ml, whereas the low responders received a cumulative dose of 11.2 ± 2.74 ml. Other laboratory values were not significantly different (data not shown). A longitudinal linear mixed effects model also revealed that change in eGFR over time changes depended on the expression of RET on the REACT product. This interaction trended toward significance (*P* = 0.09).

### Serious Adverse Events

SAEs were common in this population due to the comorbidities of advanced diabetic kidney disease (DKD) and metabolic syndrome but were similar to other historical CKD trials. No SAEs were associated with the biopsies and REACT injections. A total of 61 SAEs occurred in 15 of 22 patients, with the 5 most common categories being cardiac, infectious, renal, respiratory, and metabolic (see Table 3). Among those with 2 REACT injections, 2 had fatal SAEs and 1 progressed to ESKD. One patient experienced squamous cell carcinoma of the lung, causing left lung collapse/postobstructive pneumonia and hypercalcemia, resulting in death 12 months post second REACT injection. An autopsy was declined by the family. A second patient had a myocardial infarction, and the third patient (screening eGFR of 33 ml/min per 1.73 m^2^ and heavy proteinuria) had rapid decline in eGFR to 20 ml/min per 1.73 m^2^ at 6 months and was placed on hemodialysis 11 months post-second REACT injection.

**Table 3:** Serious adverse events by system organ class and preferred terminology based on the medical dictionary for regulatory activities. N = 15 of 22 patients

System organ class N = 15	Preferred terminology
Cardiac disorders (*n =* 16)	
Acute myocardial infarction	6
Atrioventricular block complete	1
Cardiac arrest	2
Cardiac failure acute	2
Coronary artery disease	3
Left ventricular failure	1
Myocardial infarction	1
Gastrointestinal disorders (*n =* 2)	
Diarrhea	1
Diverticulum	1
Hepatobiliary disorders (*n =* 1)	
Cholecystitis acute	1
Infections and infestations (*n =* 12)	
Cellulitis	2
Cholecystitis infective	1
Clostridium difficile infection	1
Coronavirus infection	1
Device related infection	1
Infected bite	1
Peritonitis	1
Pneumonia	2
Sepsis	1
Staphylococcal infection	1
Injury (*n =* 2)	
Fall	1
Patella fracture	1
Metabolism and nutrition disorders (*n =* 5)	
Dehydration	1
Hypercalcemia	1
Hyperkalemia	3
Musculoskeletal disorders (*n =* 1)	
Meniscal degeneration	1
Neoplasms benign/malignant (*n =* 1)	
Squamous cell carcinoma of lung	1
Nervous system disorders (2)	
Cerebrovascular accident	1
Syncope	1
Psychiatric disorders (1)	
Hallucination, visual	1
Renal and urinary disorders (9)	
Acute kidney injury	8
End-stage renal disease	1
Respiratory, thoracic, and mediastinal disorders (6)	
Acute respiratory failure	1
Chronic obstructive pulmonary disease	1
Pneumothorax	1
Respiratory distress	1
Respiratory failure	2
Vascular disorders (3)	
Aortic stenosis	1
Deep vein thrombosis	1
Hematoma	1
Total	61

### Cell Protein Marker Analysis in the REACT Bioactive Product (SRCs)

**Figure 2:**
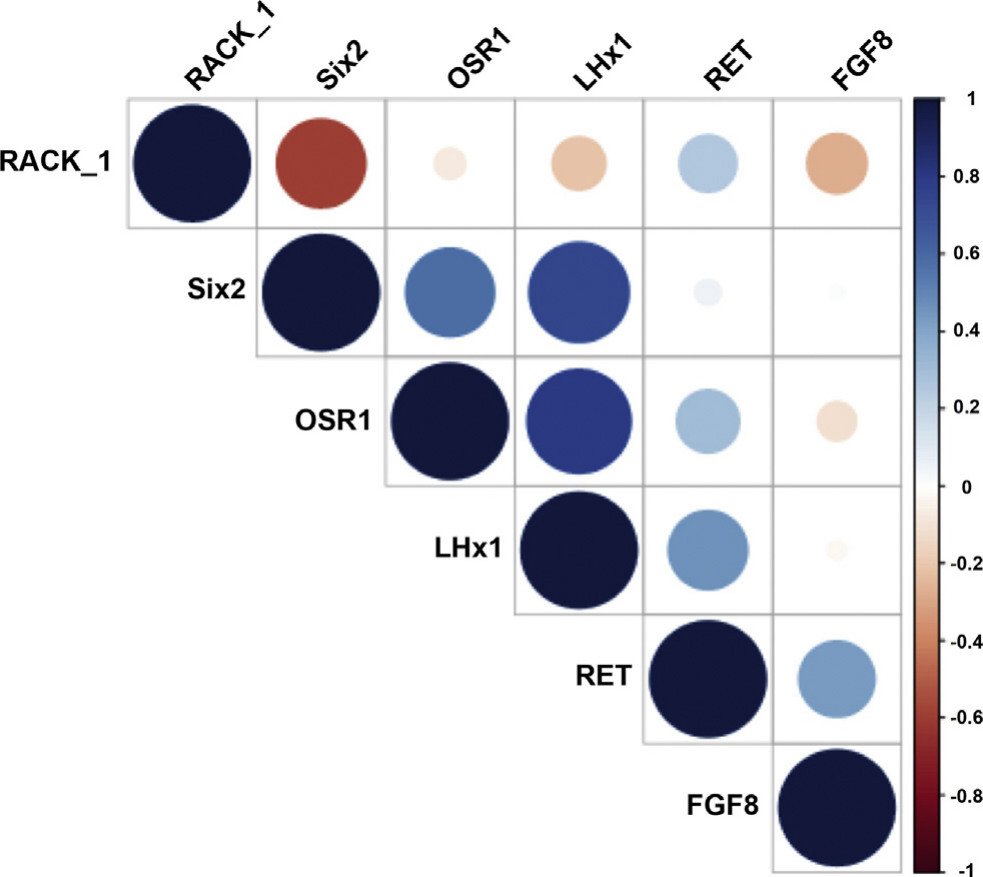
Correlations between the positivity of cell markers of the SRCs. A dark blue represents a correlation coefficient of +1, and a dark red a correlation coefficient of −1. The correlations between LHx1 and Six2 and OSR1 indicated strongest and RACK_1 weakest positivity. SRC, selected renal cell.

Results are summarized in Table 4. Correlation of various markers on FACS analysis revealed evidence for co-expression (Figure 2). Patients with a positive post-injection slope had a greater expression of RET with an average expression of 47.6% in the high/moderate group versus 20.6% in the low group (*P* = 0.027). All other cell markers were not different among patients with a positive versus a negative slope after the first injection. There was also a significant positive correlation between Six2 and RET (*P* = 0.0497). Correlation between various cell markers is depicted in Figure 2.

**Table 4:** Compiled data for fluorescent activated cell sorting analysis

Study parameter	Six 2	OSR1	LHx1	RET	FGF8	RACK-1	Nephrin	Podocin
Number of values	19	22	22	22	18	11	10	5
Minimum	0.1200	43.77	0.8700	1.260	0.01000	80.50	68.55	95.12
25% Percentile	0.6200	65.05	10.92	2.865	0.2225	91.00	78.99	95.42
Median	**1.900**	**75.72**	**32.29**	**16.53**	**1.015**	**93.70**	**89.43**	**97.46**
75% Percentile	9.500	93.38	91.93	56.12	4.403	99.00	95.47	97.71
Maximum	30.70	99.10	99.10	78.38	58.50	99.40	98.53	97.74
Range	30.58	55.33	98.23	77.12	58.49	18.90	29.98	2.620
Mean	5.929	76.88	44.59	29.18	6.980	93.63	86.79	96.74
SD	8.207	17.17	38.01	28.83	15.50	5.647	9.861	1.232
SEM	1.883	3.661	8.104	6.148	3.652	1.703	3.118	0.5508

### VEGF-A Analysis

VEGF-A was absent in negative control media using unconditioned serum-free renal cell growth media, but VEGF-A was detectable in SRC-conditioned, serum-free renal cell growth media (range 4.32–7.39 ng/ml) for up to 4 months.

## Discussion

Cell-based therapies may have the potential to modulate disease stability and offer new therapeutic options for CKD. In this cohort with moderate/severe type 2 D-CKD, percutaneously injected REACT into their kidney cortex resulted in a statistically significant improvement in eGFR decline, suggesting stabilization of renal function. eGFR, the most widely used parameter of kidney function,^24^ improved in the moderate/high responders with a positive regression line slope. Clinical/laboratory findings suggest that the SRCs in REACT may stabilize renal function. We are unaware of any studies that have revealed significantly sustained improvement of eGFR in type 2 D-CKD patients beyond our own phase I data.^25^ Importantly, in these patients, 2 injections were used, a change from our phase I trial.

All patients tolerated all procedures. This contrasts with the SAEs noted in our phase I safety trial, where REACT injections were performed by surgical laparoscopy and general anesthesia.^25^ There were 2 patients who had SAEs unrelated to the procedures, preventing a second REACT injection. The SAE profile in this high-risk population is comparable with that reported in similar CKD trials.^19^^,^^26^

We broadly witnessed 3 types of renal function responses, namely high responders (*n =* 4) with a substantial improvement of eGFR and a posttreatment slope >+2, moderate responders (*n =* 3) with a slope between zero and +2, and low responders (*n =* 15) with a slope < 0 ml/min per 1.73 m^2^. Overall, the eGFR slope became less negative in all patients, suggesting kidney function stabilization, while patients with a negative slope still had some improvement. Of note, those with a positive slope had a higher eGFR at entry. However, the number of participants is low, and more patients need to be analyzed to justify any conclusions about whether a higher entry eGFR should be favored.

Blood urea nitrogen, potassium, Hgb, and biomarkers of kidney osteodystrophy did not change. By contrast, we observed slight worsening of log parathyroid hormone and log UACR, although the magnitude of these changes may not be clinically relevant and could not be adjusted because of the small number of participants. Glycated Hgb level was not a factor in eGFR recovery. Blood pressure was unchanged except in 1 patient who received only 1 REACT injection.

REACT is composed principally of selected kidney epithelial cells from the nephron, including proximal tubules, glomeruli, and smaller numbers of other cells.^15^ We have previously revealed that SRC implantation in rodents can induce neo kidney-like tissue histologic changes at the site of implantation.^10^^,^^11^^,^^16^ In this study, we did not trace the cells. However, using MR imaging in 2 rodent models, implanted SRCs were detectable 7 days and up to 6 months postinjection into the kidney tissue.^10^^,^^11^ Our novel data include compositional analysis of REACT, revealing the presence of cellular markers associated with the earliest stages of nephron development. Unfortunately, institutional review board concerns prevented us from performing follow-up kidney biopsies to assess histology. However, the improvements in eGFR observed, the analysis of the REACT product, and evidence from animal models suggest a similar mechanism may be occurring.

We provide data on the SRC protein expression, including SIX2, Osr1, RET, LHx1, FGF8, Rack1, Nephrin (NPHS1), and Podocin (NPHS2) using FACS analysis, and moderate/high responders were found to have more RET. Osr1 was consistently expressed in the SRCs and is the earliest marker of the intermediate mesoderm that form the gonads and kidneys.^27^ This expression is not essential for the formation of intermediate mesoderm but is essential for the differentiation toward renal and gonadal structures.^27^ Progenitor cells are descendants of stem cells that then further differentiate to create specialized cell types. Therefore, we hypothesize that the REACT product does contain renal progenitor cells.

It is unclear whether the difference in the expression of RET is responsible for the stronger effect on eGFR. The FACS analysis suggests that membrane-bound and nuclear transcription factors representing ureteric bud, cap mesenchyme, and glomerular lineages were present in each patient’s REACT product. The ureteric bud is an epithelial tube that arises from the nephric duct and branches repetitively to give rise to the kidney collecting duct system, while also generating inductive signals with the cap mesenchyme that directs mesenchymal-to-epithelial transformation and promotion of nephrogenesis by the surrounding metanephric mesenchyme cells.^28^

SIX2 defines and regulates a multipotent self-renewing nephron progenitor population throughout mammalian kidney development, as SIX2-expressing cells give rise to all cell types of the main body of the nephron, during all stages of nephrogenesis.^29^ During normal development, SIX2 expression and SIX2+ nephron progenitor cells in the cap mesenchyme both rapidly disappear after birth.^30^ However, in a rat model, preserved SIX2 expression was found after partial nephrectomy in a 1-day-old neonatal rats, resulting in neonephrogenesis.^31^ The presence of SIX2 in 19 of 22 patients and a companion CM marker, OSR1, revealed lineage to the cap mesenchyme progenitor line in the SRCs.

Moreover, the LIM-class homebox transcription factor LHx1 is expressed early in the intermediate mesoderm and is one of the first genes to be expressed in the nephric mesenchyme. LHx1 is required for the specification of the renal progenitor cell field.^32^ Using an explant culture system to induce kidney tissue, Cirio *et al.*^32^ revealed that expression of genes from both proximal and distal kidney structures is affected by the absence of LHx1.

A key signal that promotes ureteric bud morphogenesis is GDNF, a protein secreted by metanephric mesenchyme cells that signals to ureteric bud cells by the rearrangement in transformation RET receptor tyrosine kinase, messaging the mesenchymal-to-epithelial cell transformation. In the ureteric bud and collecting ducts, RET receptor tyrosine kinase, GDNF, and its co-receptor, GDNF family receptor α 1, initiate a signaling cascade that triggers the growth of RET-positive cells from the nephric duct toward GDNF cells of the metanephric mesenchyme.^33^ Detection of RET-positive cells in our SRC population suggests the presence of a cell population capable of responding to extracellular signaling and giving rise to neo kidney-like tissue. There was significantly higher RET-positive cell expression in the patients with a positive eGFR slope after the first injection. With embryologic nephrogenesis, RET signaling, by Etv4 and Etv5, promotes competitive cell rearrangements in the nephric duct, in which the cells with the highest level of RET signaling preferentially migrate to form the first ureteric bud tip.^33^^,^^34^ RET signaling in ureteric bud cells is key for controlling cell movement, cell clustering, and ureteric bud formation during nephrogenesis.^34^ Whether RET expression is indeed the most important factor requires further evaluation.

Functional evaluation of SRCs provides evidence that activation of certain key developmental pathways may represent a potential mechanism of regenerative bioactivity. Molecular genetics of these developmental pathways and critical proteins that mediate nephrogenesis and their potential relevance to regeneration have been described.^17^ Nephrogenesis is a dynamic cellular migration/differentiation, induced by crosstalk signaling in resident cells. It is an integral part of nephron development.

Nephrin and podocin are markers at the slit diaphragm and podocyte pedicels, respectively, and imply glomerulus lineage at the Glomerular Basement Membrane (GBM). Nephrin and the GBM form the filtration barrier , critical to repel albumin and other macromolecules from entering the Bowman’s capsule, preventing epithelial inflammatory change. Glomerulogenesis is divided into the following stages: vesicle, comma- and S-shaped, glomerular capillary loop stage, and mature glomerulus,^35^ and we have revealed these stages on histology in our animal models of SRCs.^16^ Animal model images closely resemble human nephrogenesis.^36^ Furthermore, the expression of cap mesenchyme,^37^, ^38^, ^39^ ureteric,^34^ and glomerular cell markers^40^^,^^41^ resembles *in utero* embryologic studies. Animal data may not completely be applicable to humans, given species evolutionary differences, but they provide a practical model. Although we have no histopathologic evidence from the patients, cell markers presented in REACT have been described as essential for nephrogenesis. The populations of SRCs contained cells responsible for nephrogenesis, and the markers analyzed represent the critical pathway for the development of the renal cortex, medullary interstitium, angioblasts, and mesangium (Table 4). These data align with our hypothesis that any improved renal function resultant from REACT injections may be due to neo kidney-like tissue development, mirroring embryonic kidney development.

In cadaveric donor-derived SRCs processed identically to REACT, we also revealed limited evidence for the expression of VEGF-A in vitro. VEGF is a proangiogenic glycoprotein in the platelet-derived growth factor family, essential for the survival, proliferation, and differentiation of endothelial cells.^42^^,^^43^ VEGF has a role in angiogenesis/vasculogenesis during embryogenesis, maintaining renal homeostasis during cell migration and is expressed throughout the life of podocytes and tubular cells.^44^, ^45^, ^46^ VEGF-A was expressed by donor SRCs, providing indirect evidence for angiogenesis promoting cell division, migration, endothelial cell survival, and vascular sprouting.^47^^,^^48^ Our data begin to reveal a putative mechanism of action for REACT, providing the cells involved in a developmental pathway that stimulates cell migration into the diseased tissue, contributing toward neo kidney-like tissue and kidney function stabilization.

This report may serve as a proof-of-concept in humans. Additional data and analysis are required to identify which patients may benefit most from REACT therapy and to provide further evidence on the mechanism of action. Larger phase III human trials of REACT are underway (http://www.prokidney.com/clinical-trials/, accessed January 30, 2022).

### Limitations

Our sample is underpowered to evaluate the impact of all covariates and bias assessment. Most patients were Caucasian and there were missing data. Our cohort is part of a larger parent study, and for this report, only 22 patients had functional SRC studies and consented for publication. Some participants had small residual cell volumes after their REACT injections, preventing full FACS analysis. There may be inherent technical limitations of the FACS analysis such as cell asynchrony. Furthermore, we only characterized the SRCs with FACS analysis after the expansion, and it is possible that this expansion process altered the results. Moreover, we did not measure CD24 and CD133 or the stem-cell specific transcription factors Oct-4 and Bml-1 and cannot confirm the presence of pluripotent stem cells responsible for formation of glomeruli as revealed by Sagrinati *et al.*^49^

The patients with a positive slope tended to have a higher entry eGFR, and although their UACR progressed slower, the number of patients is small. We were unable to study the impact of pretreatment eGFR slope over 3 months in several patients based on the study protocol. In some patients, we had a much shorter preinjection slope (see Figure 1) which may explain why some patients had a positive slope before injection, given intrapatient variability of sCr. Nonetheless, the sustained increase in eGFR in 7 of 22 patients is remarkable. We also do not have exogenous benchmark GFR measurements, such as iohexol. However, in our studies with 70% nephrectomized canines, SRCs increased iohexol clearance.^15^

Although usable SRCs could produce REACT from all biopsies, there is a possibility that the yield of usable cells from the biopsy might have been lower from patients with lower eGFR, as much of their kidney tissue could have more fibrosis, affecting FACS positivity.^7^^,^^50^ Additional studies are needed to confirm our findings and are underway.^13^^,^^14^^,^^19^ Moreover, we must analyze more SRCs for the various expressions of cell line markers to determine minimum requirements for the injectable product. Further analyses with genome-wide transcriptional and epigenetic profiling of SRCs may lend support to complex developmental pathways as mechanisms of action for regenerative CKD therapies. Finally, data from animal studies cannot necessarily be extrapolated into humans and only *in vitro* data are presented to support the hypothesis that the GFR stabilization may be due to neo nephrogenesis.

### Summary

Mechanisms of action and potency of cell therapy products are complex and recognized by regulatory agencies.^51^ Autologous homologous CKD cell therapies are equally complex due to multiple cell types and the many potential functional effects at the various levels in the nephron, spanning from the glomerulus to the distal convoluted tubule and collecting duct.

We have revealed that the SRCs in the REACT product evolved from preclinical trials^12^ and successfully stabilized kidney function in adults with type 2 D-CKD (abstract 3611676 “Renal autologous cell therapy (REACT) for type 2 D-CKD: preliminary results with renal cortex implantation” for the American Society of Nephrology Renal Week 2021). On the basis of preclinical animal studies and this preliminary evidence, it is hypothesized that SRCs in the REACT product may function in part by promoting the assembly of progenitor cells lines, through secretion of pro-angiogenic factors, such as VEGF-A, repairing effete nephrons and possibly producing neo kidney-like tissue.

In conclusion, our trial findings suggest that the SRCs of the REACT product may initiate neo kidney-like tissue development to stabilize and improve kidney function and halttype 2 D-CKD progression. There may be benefits for a higher entry eGFR, multiple doses of REACT, and/or personalized dosing intervals to accommodate disease progression; however, the best treatment regimen remains to be elucidated.

## Disclosure

JS, DJ, JWL, JB, RP, EB, and TB are employees of ProKidney and received wages. GF and MF received consulting fees from ProKidney. GF, JL, JB, and MF received fees for advisory roles at ProKidney.

## Supplementary Material

Supplementary File (PDF)

**Appendix 1.** Composition of cap mesenchyme, ureteric bud, and glomerular cell lines using cell biomarkers.

**STROBE Checklist.** Strobe Checklist for prospective cohort study.
