# Supplementary material for: Renal Autologous Cell Therapy to Stabilize Function in Diabetes-Related Chronic Kidney Disease: Corroboration of Mechanistic Action With Cell Marker Analysis
Source: Kidney Int Rep. 2022 Apr 21;7(7):1619–29. doi: 10.1016/j.ekir.2022.04.014 (PMC9263255; doi:10.1016/j.ekir.2022.04.014)
Supplement: Supplementary File (PDF) [file mmc1.pdf]

Appendix 1: **Composition of cap mesenchyme, ureteric bud and glomerular cell lines using cell biomarkers**

Briefly, the three main cell constituents key to kidney development include the cap mesenchyme, ureteric bud , and glomerular tuft.[50] For the purpose of this manuscript, we focused on proteins encoded by six proteins that are differently expressed by the progenitor cell lines of these tissues in the kidney. These were as follows:

Cap mesenchyme cell markers:

Sine oculis homeobox homolog (SIX) family proteins, including that encoded by the *SIX2* gene, are involved in the development of several organs, including the kidney. During kidney development, *SIX2* maintains cap mesenchyme multipotent nephron progenitor cells in an undifferentiated state by opposing the inductive signals emanating from the ureteric bud and cooperates with WNT9B to promote renewing progenitor cells proliferation.[35] Mutations of SIX2 have been associated with kidney hypodysplasia.[51]

Protein odd-skipped related 1 (OSR1) is a zinc-finger transcription factor that, in humans, is encoded by the *OSR1* gene found on chromosome 2 (2p24.1) and is involved in the development of the kidney.[36]

The LHX-1 gene encodes the **LIM homepox 1 protein (LHX1)**, which is a transcription factor important for the control of differentiation and development of neural and lymphoid cells as well as the kidney and urogenesis and is required for normal organogenesis.[37]

Ureteric bud cell marker:

Ret signaling promotes branching morphogenesis of the ureteric bud. Ret-expressing progenitor cells proliferate at the ureteric bud tips.[38]

Fibroblast growth factor 8 (FGF8) coordinates tissue elongation and cell epithelization during early kidney tubulogenesis.[52]

Scaffolding proteins:

The **Receptor for Activated C Kinase 1 (RACK1)** is a member of the tryptophan-aspartate repeat (WD-repeat) family of proteins.[53] These proteins represent critical pathways for the development of the kidney cortex, medullary interstitium, angioblasts and mesangium.

Glomerular cell markers:

Nephrin and **podocin** are podocyte proteins encoded by the *NPHS1* and *NPHS2* gene, respectively. Podocyte foot processes attach themselves to the glomerular capillaries at the glomerular basement membrane forming intercellular junctions that form slit diaphragm filtration barriers (two nephrin proteins from each of the two podocytes interlink) and represent the most important filtration barrier for protein.[39] Podocin is a 383 amino acid membrane protein of the band-7-stomatin family with a transmembrane domain forming a hairpin structure, and two cytoplasmic ends at the N- and C-terminus, which interact with the cytosolic tail of nephrin.[40]

STROBE Statement—checklist of items that should be included in reports of observational studies

	Item No.	Recommendation	Page No.	Relevant text from manuscript
**Title and abstract**	1	(*a*) Indicate the study's design with a commonly used term in the title or the abstract	2	This clinical trial
		(*b*) Provide in the abstract an informative and balanced summary of what was done and what was found	2	See abstract
**Introduction**				
Background/rationale	2	Explain the scientific background and rationale for the investigation being reported	4-5	See introduction
Objectives	3	State specific objectives, including any prespecified hypotheses	5	This is a proof-of-concept clinical trial suggesting that SRCs therapy may result in kidney function stabilization through neo kidney-like tissue
**Methods**				
Study design	4	Present key elements of study design early in the paper	5-8	See methods section
Setting	5	Describe the setting, locations, and relevant dates, including periods of recruitment, exposure, follow-up, and data collection	5	conducted in major teaching hospitals in the US
Participants	6	(*a*) *Cohort study*—Give the eligibility criteria, and the sources and methods of selection of participants. Describe methods of follow-up<br>*Case-control study*—Give the eligibility criteria, and the sources and methods of case ascertainment and control selection. Give the rationale for the choice of cases and controls<br>*Cross-sectional study*—Give the eligibility criteria, and the sources and methods of selection of participants	5-6	See study population
		(*b*) *Cohort study*—For matched studies, give matching criteria and number of exposed and unexposed<br>*Case-control study*—For matched studies, give matching criteria and the number of controls per case	N/A	
Variables	7	Clearly define all outcomes, exposures, predictors, potential confounders, and effect modifiers. Give diagnostic criteria, if applicable	6-7	See clinical and laboratory data

Data sources/ measurement	8*	For each variable of interest, give sources of data and details of methods of assessment (measurement). Describe comparability of assessment methods if there is more than one group	6-7	See methods section
Bias	9	Describe any efforts to address potential sources of bias	8	See statistics section
Study size	10	Explain how the study size was arrived at	8	See statistics section

Continued on next page

Quantitative variables	11	Explain how quantitative variables were handled in the analyses. If applicable, describe which groupings were chosen and why	8	See statistics section
Statistical methods	12	(*a*) Describe all statistical methods, including those used to control for confounding	8	See statistics section
		(*b*) Describe any methods used to examine subgroups and interactions	8	See statistics section
		(*c*) Explain how missing data were addressed	8	See statistics section
		(*d*) *Cohort study*—If applicable, explain how loss to follow-up was addressed<br>*Case-control study*—If applicable, explain how matching of cases and controls was addressed<br>*Cross-sectional study*—If applicable, describe analytical methods taking account of sampling strategy	N/A	
		(*e*) Describe any sensitivity analyses	Not done	See statistics section
**Results**				
Participants	13*	(a) Report numbers of individuals at each stage of study—eg numbers potentially eligible, examined for eligibility, confirmed eligible, included in the study, completing follow-up, and analysed	8	See patients' section of results
		(b) Give reasons for non-participation at each stage	8	See patients' section of results
		(c) Consider use of a flow diagram	N/A	
Descriptive data	14*	(a) Give characteristics of study participants (eg demographic, clinical, social) and information on exposures and potential confounders	8 + table 1	See results section
		(b) Indicate number of participants with missing data for each variable of interest	8-11	See results section
		(c) *Cohort study*—Summarise follow-up time (eg, average and total amount)	9	The median follow up was 24.3 months (IQR 18.8, 27.7).
Outcome data	15*	*Cohort study*—Report numbers of outcome events or summary measures over time	8-11	See results section
		Case-control study—Report numbers in each exposure category, or summary measures of exposure		
		Cross-sectional study—Report numbers of outcome events or summary measures		
Main results	16	(*a*) Give unadjusted estimates and, if applicable, confounder-adjusted estimates and their precision (eg, 95% confidence interval). Make clear which confounders were adjusted for and why they were included	8-11	See results section
		(*b*) Report category boundaries when continuous variables were categorized	N/A	
		(*c*) If relevant, consider translating estimates of relative risk into absolute risk for a meaningful time period	N/A	

Continued on next page

Other analyses	17	Report other analyses done—eg analyses of subgroups and interactions, and sensitivity analyses	9	Patients with a post-injection eGFR slope >0 vs. <0
**Discussion**				
Key results	18	Summarise key results with reference to study objectives	11-12	See first two paragraphs of discussion
Limitations	19	Discuss limitations of the study, taking into account sources of potential bias or imprecision. Discuss both direction and magnitude of any potential bias	16-17	See limitations section
Interpretation	20	Give a cautious overall interpretation of results considering objectives, limitations, multiplicity of analyses, results from similar studies, and other relevant evidence	17-18	See summary section
Generalisability	21	Discuss the generalisability (external validity) of the study results	16-17	See limitations section
**Other information**				
Funding	22	Give the source of funding and the role of the funders for the present study and, if applicable, for the original study on which the present article is based	22	Line 560

*Give information separately for cases and controls in case-control studies and, if applicable, for exposed and unexposed groups in cohort and cross-sectional studies.

Note: An Explanation and Elaboration article discusses each checklist item and gives methodological background and published examples of transparent reporting. The STROBE checklist is best used in conjunction with this article (freely available on the Web sites of PLoS Medicine at http://www.plosmedicine.org/, Annals of Internal Medicine at http://www.annals.org/, and Epidemiology at http://www.epidem.com/). Information on the STROBE Initiative is available at www.strobe-statement.org.
